# Identifying causal genes for stroke via integrating the proteome and transcriptome from brain and blood

**DOI:** 10.1186/s12967-022-03377-9

**Published:** 2022-04-21

**Authors:** Bang-Sheng Wu, Shu-Fen Chen, Shu-Yi Huang, Ya-Nan Ou, Yue-Ting Deng, Shi-Dong Chen, Qiang Dong, Jin-Tai Yu

**Affiliations:** 1 Department of Neurology and Institute of Neurology, Huashan Hospital, State Key Laboratory of Medical Neurobiology and MOE Frontiers Center for Brain Science, Shanghai Medical College, Fudan University, National Center for Neurological Disorders, Shanghai, China; 2 Department of Neurology, Qingdao Municipal Hospital, Qingdao University, Qingdao, China

**Keywords:** Stroke, Proteome-wide association study, Transcriptome-wide association study, Mendelian randomization, Bayesian colocalization

## Abstract

**Background:**

Genome-wide association studies (GWAS) have revealed numerous loci associated with stroke. However, the underlying mechanisms at these loci in the pathogenesis of stroke and effective stroke drug targets are elusive. Therefore, we aimed to identify causal genes in the pathogenesis of stroke and its subtypes.

**Methods:**

Utilizing multidimensional high-throughput data generated, we integrated proteome-wide association study (PWAS), transcriptome-wide association study (TWAS), Mendelian randomization (MR), and Bayesian colocalization analysis to prioritize genes that contribute to stroke and its subtypes risk via affecting their expression and protein abundance in brain and blood.

**Results:**

Our integrative analysis revealed that *ICA1L* was associated with small-vessel stroke (SVS), according to robust evidence at both protein and transcriptional levels based on brain-derived data. We also identified *NBEAL1* that was causally related to SVS via its cis-regulated brain expression level. In blood, we identified 5 genes (*MMP12*, *SCARF1*, *ABO*, *F11*, and *CKAP2*) that had causal relationships with stroke and stroke subtypes.

**Conclusions:**

Together, via using an integrative analysis to deal with multidimensional data, we prioritized causal genes in the pathogenesis of SVS, which offered hints for future biological and therapeutic studies.

**Supplementary Information:**

The online version contains supplementary material available at 10.1186/s12967-022-03377-9.

## Introduction

As the second-leading cause of death globally, stroke contributed to 6.55 million people’s deaths in 2019 with disability-adjusted life years (DALYs) increased steadily [1], which warranted novel therapies for treatment of stroke. Although there are many risk factors that are simultaneously associated with stroke, the causal genes responsible for stroke has remained unexplored. Thus, efforts are still required to identify the key molecular signatures in the pathogenesis of stroke to provide a fundamental theory for treatment.

With the development of high-throughput sequencing technology, genome-wide association studies (GWASs) have identified numerous loci associated with stroke [2]. Despite several efforts, the underlying mechanism attributed to stroke risk is elusive, which hinders the translation from identified risk loci to clinical therapy.

Recently, large-scale quantitative trait loci (QTL) data were produced to establish the association between genotype with protein abundance (pQTL) and gene expression (eQTL) [3, 4], which led to continuous emergent of statistical methods facilitating the integration of the multidimensional data [5]. Proteome-wide association studies (PWASs) have been recently used to find candidate genes whose protein abundances are associated with Alzheimer’s disease and depression [6, 7]. Similarly, transcriptome-wide association studies (TWASs) have been applied in the association analyses between gene expression and phenotypes [8]. Besides, Mendelian randomization (MR) and Bayesian colocalization analysis were also widely used to identify candidate genes via integrating QTL and disease GWAS data [9, 10]. Mendelian randomization, which simulates a natural randomized controlled trial (mutations are randomly assigned to gametes during meiosis), can provide causal inference under the three core assumptions [11, 12]. Bayesian colocalization analysis calculates the probability that two traits share a causal genetic variant [13]. Altogether, integrating GWAS data with these multidimensional QTL data shall help prioritize specific pathways and candidate genes to discriminate the potential genes accounting for the pathogenesis of stroke.

Moreover, a previous study pointed out that mononuclear cells in peripheral could be used as a biomarker in ischemic stroke [14]. Thus, in addition to investigating the directly related brain tissue, we also applied our analysis in blood to reach a more comprehensive understanding of stroke pathogenesis.

**Fig. 1:**
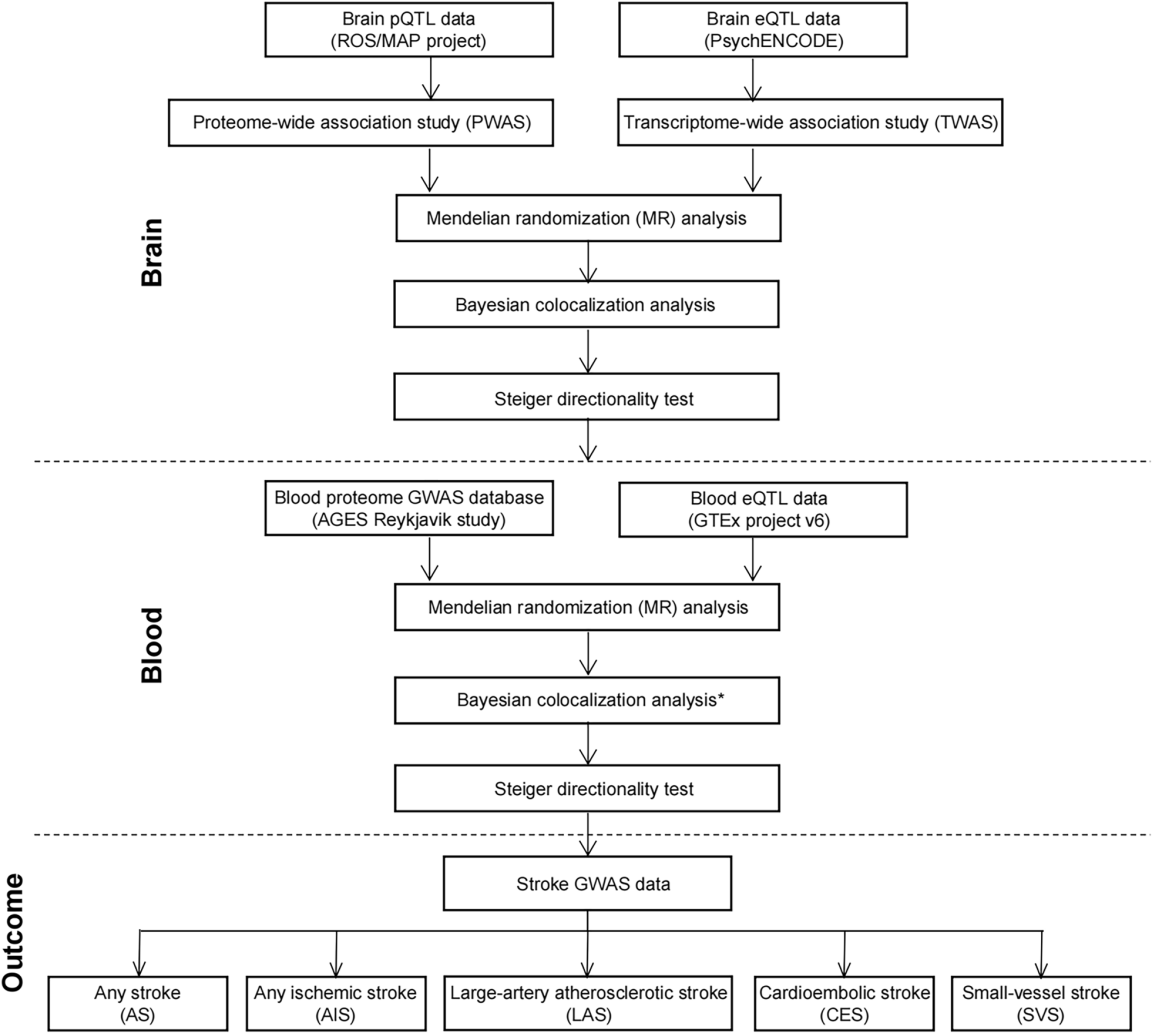
Flowchart of the study. First, we leveraged p/eQTL data derived from the brain tissue with stroke GWASs to conduct a PWAS/TWAS analysis followed by Mendelian randomization (MR), Bayesian colocalization analysis, and Steiger directionality test. Then, we applied similar analysis framework to integrate the proteomic data and eQTL data derived from blood with stroke GWASs. * indicated Bayesian colocalization analysis was only performed for eQTL data

In this study, we conducted an integrative analysis to identify candidate genes for stroke and stroke subtypes by combing brain-derived and blood-derived multi-omics data with genetic data. The overall analysis pipeline is shown in Fig. 1. First, we utilized pQTL and eQTL data derived from brain tissues and GWAS findings of stroke to perform PWAS and TWAS separately. Then, MR, Bayesian colocalization, and Steiger filtering analysis were leveraged to detect the causal relationship between stroke and genomic architecture-associated protein or transcription levels. Second, the integrative analysis was mapped on blood-derived multi-omics data to test the consistency between brain and blood. Our study prioritized candidate genes underlying complex forms of stroke, which could serve as potential treatment targets.

## Methods

### Stroke GWAS data

The current study utilized stroke GWAS summary datasets obtained from the MEGASTROKE consortium [2]. The stroke GWAS summary datasets used in the main analysis were restricted to European participants, including data for 446,696 European descent individuals (40,585 any stroke cases and 406,111 controls). The ancestry-specific meta-analyses were also conducted for four stroke subtypes, including any ischemic stroke (AIS; 34,217 cases), large artery stroke (LAS; 4,373 cases), cardioembolic stroke (CES; 7,193 cases), and small vessel stroke (SVS; 5,386 cases).

### Human brain proteomic and transcriptomic data

We analyzed the proteomes of 400 postmortem brain samples with dorsolateral prefrontal cortex (dPFC) from the ROS/MAP [3]. In more details, the digested peptides were labeled with isobaric tandem mass tag (TMT) and subjected to liquid chromatography coupled to mass spectrometry (LC–MS) for sequencing. Genotypes were derived from either whole-genome sequencing or genome-wide genotyping by either the Illumina OmniQuad Express or Affymetrix GeneChip 6.0 platforms [15]. The quality control process was described in the primary study. Finally, 376 subjects with both proteomic and genetic data passed the quality control for the PWAS.

The eQTL dataset was from the PsychENCODE Consortium covering 1,129,652 eQTLs of 11,120 genes from the prefrontal cortex (PFC) (n = 1387) [4]. We only included the data of SNPs within 1 MB window around each gene. Genotypes were derived either from genome-wide single nucleotide polymorphisms (SNP) arrays or whole genome sequencing.

### Human blood proteomic and transcriptomic data

The serum proteomic data was derived from a large population-based study (AGES Reykjavik study; n = 5457) [16]. The AGES Reykjavik study consisting of predominantly European individuals older than 65 years of age, whose phenotype and genotype information were available. The Slow-Off rate Modified Aptamer (SOMAmer), a proteomic profiling platform, was used to determine the serum levels of 4137 human proteins.

The whole-blood eQTL data was derived from the Genotype-Tissue Expression (GTEx) version 6 database (n = 338) [17]. The gene expression data was obtained using paired-end RNA-seq (Illumina TruSeq; Illumina Inc) and the genotype data was from whole-genome sequencing. Full descriptions of donor registration, consent process, biological sample acquisition methods, sample attachment and histopathological examination procedures are available on the official GTEx website [18].

### Statistical analysis

Functional Summary-based Imputation (FUSION) software was used to estimate protein weights using proteomic and genetic data from ROS/MAP [8]. Briefly, a linkage disequilibrium (LD) reference panel was used to minimize the influence of LD on the estimated test statistics [8]. Then, the SNP-based heritability for each gene was estimated and we used FUSION to compute the effect of SNPs with significant heritability (*P* < 0.01) on protein abundance using multiple predictive models, including top1, blup, lasso, enet and bslmm [8]. The weights of protein were obtained from the most predictive model. The protein weights used in the current study were derived from [19] and the expression weights were derived from transcriptomic data generated from dPFC (CommonMind Consortium; n = 452) [20, 21]. Later, we used FUSION to combine the genetic effect of stroke (stroke GWAS z-score) with the protein or expression weights by calculating the linear sum of z-score × weight for the independent SNPs at the locus to perform the PWAS or TWAS. Bonferroni-corrected P value threshold was used to reduce the instance of a false positive. We also calculated the P value adjust for false discovery rate (FDR) using Benjamini-Hochberg (BH) method.

Mendelian randomization [22] used the SNPs as an instrumental variable (IV) to infer the causal relationship between exposure and outcome and the quantitative trait loci data can be integrated to investigate the causal gene of disease [23]. Genome-wide significant (*P* < 5 × 10^–08^) SNPs were selected and followed LD clumping to obtain independent SNPs (R^2^ > 0.001). Then the exposure (QTL data) and outcome (stroke GWAS data) data were harmonized according to the same effect alleles. When only a single independent QTL was available, the Wald ratio was used to estimate the causality of exposure to outcome. Where more than one SNP was available, the inverse-variance weighted (IVW) [24] method was used to combine the ratios of SNP-exposure to SNP-outcome in a fixed-effects meta-analysis or random-effects meta-analysis. A Bonferroni-corrected threshold of *P* < 0.05/number of genes analyzed was set for multiple comparison. Besides, the Steiger filtering method [25] was employed to test if the causal direction between the hypothesized exposure and outcomes was valid using the directionality_test() function in “TwoSampleMR” package. The Mendelian randomization analysis was performed using the “TwoSampleMR” version 0.5.5 in R version 4.0.

We performed Coloc, a Bayesian test for colocalization, to evaluate the probability of stroke risk loci and p/eQTL shared by a same causal signal [13]. We assigned the default prior probabilities for a SNP being associated with stroke (p1 = 1 × 10^−4^), a SNP is a significant QTL (p2 = 1 × 10^−4^) and for a SNP being associated with both traits (p12 = 1 × 10^–5^) [26]. “coloc.abf” function in coloc R package (version 3.2.1) was used to perform colocalization on the shared SNPs from both the QTL and stroke datasets, and we focused on genes that met the Bonferroni-corrected P value threshold in previous MR analysis. Five mutually exclusive hypotheses was tested: (1) no causal SNP is found for either trait (H0); (2) only trait 1 has a causal SNP (H1); (3) only trait 2 has a causal SNP (H2); (4) both traits have a causal SNP, but the two causal SNPs are different (H3); (5) both traits have a causal SNP, and share the same SNP (H4) [26]. We mainly focused on the last hypothesis H4 and posterior probability (PP) was used to quantify support for H4 (denoted as PPH4). We defined a strong evidence of colocalization at PPH4 ≥ 0.75 [27].

## Results

### PWAS identified 6 genes associated with stroke

**Fig. 2:**
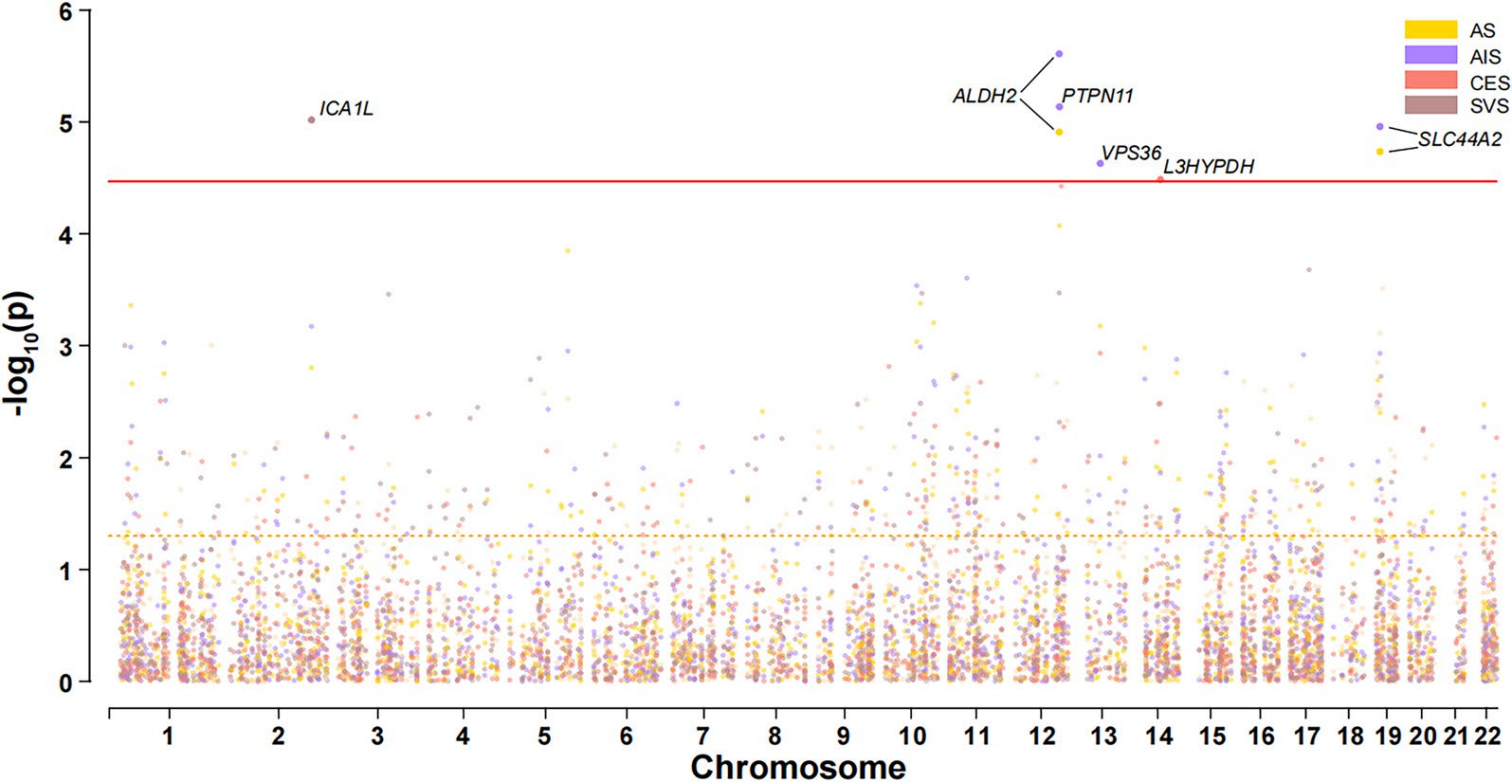
The Manhattan plot for the PWAS of stroke and stroke subtypes. The Manhattan plot shows the genes identified using PWAS for AS, AIS, LAS, CES, and SVS, respectively. The red line indicates the significant threshold for Bonferroni correction (*P* < 0.05/1468). The orange dash line indicates the nominal significance level (*P* < 0.05)

We performed a PWAS of stroke by integrating stroke GWAS results with human brain proteomes using the FUSION pipeline [6]. The PWAS identified 6 genes whose brain protein abundances were associated with stroke (Bonferroni-corrected threshold of *P* < 0.05/number of genes analyzed) (Fig. 2 and Table 1). The protein abundances of *ALDH2* (AS: Z-score: 4.372, *P* = 1.23 × 10^−5^; AIS: Z-score: 4.712, *P* = 2.46×10^−6^) and *SLC44A2* (AS: Z-score: − 4.284, *P* = 1.84×10^−5^; AIS: Z-score: − 4.396, *P* = 1.10 × 10^−5^) were both associated with AS and AIS, and the protein abundances of *PTPN11* (Z-score: − 4.484, *P* = 7.31 × 10^-6^) and *VPS36* (Z-score: − 4.229, *P* = 2.35 × 10^−5^) were also associated with AIS (Additional file 1: Table S1 and S2). However, no significant association was found for LAS (Additional file 1: Table S3). For CES and SVS, the protein abundances of *L3HYPDH* (CES: Z-score: − 4.153, *P* = 3.28 × 10^−5^) and *ICA1L* (SVS: Z-score: − 4.426, *P* = 9.60 × 10^−6^) were associated with them respectively (Additional file 1: Table S4 and S5).

**Table 1:** Candidate genes in brain proteomes associated with stroke and stroke subtypes using PWAS

Stroke subtype	Gene	CHR	Z-score	P-value
AS	*ALDH2*	12	4.372	1.23 × 10^–5^
	*SLC44A2*	19	− 4.284	1.84 × 10^–5^
AIS	*ALDH2*	12	4.712	2.46 × 10^–6^
	*PTPN11*	12	− 4.484	7.31 × 10^–6^
	*VPS36*	13	− 4.229	2.35 × 10^–5^
	*SLC44A2*	19	− 4.396	1.10 × 10^–5^
LAS	–			
CES	*L3HYPDH*	14	− 4.153	3.28 × 10^–5^
SVS	*ICA1L*	2	− 4.426	9.60 × 10^–6^

### TWAS identified 7 genes associated with stroke

**Fig. 3:**
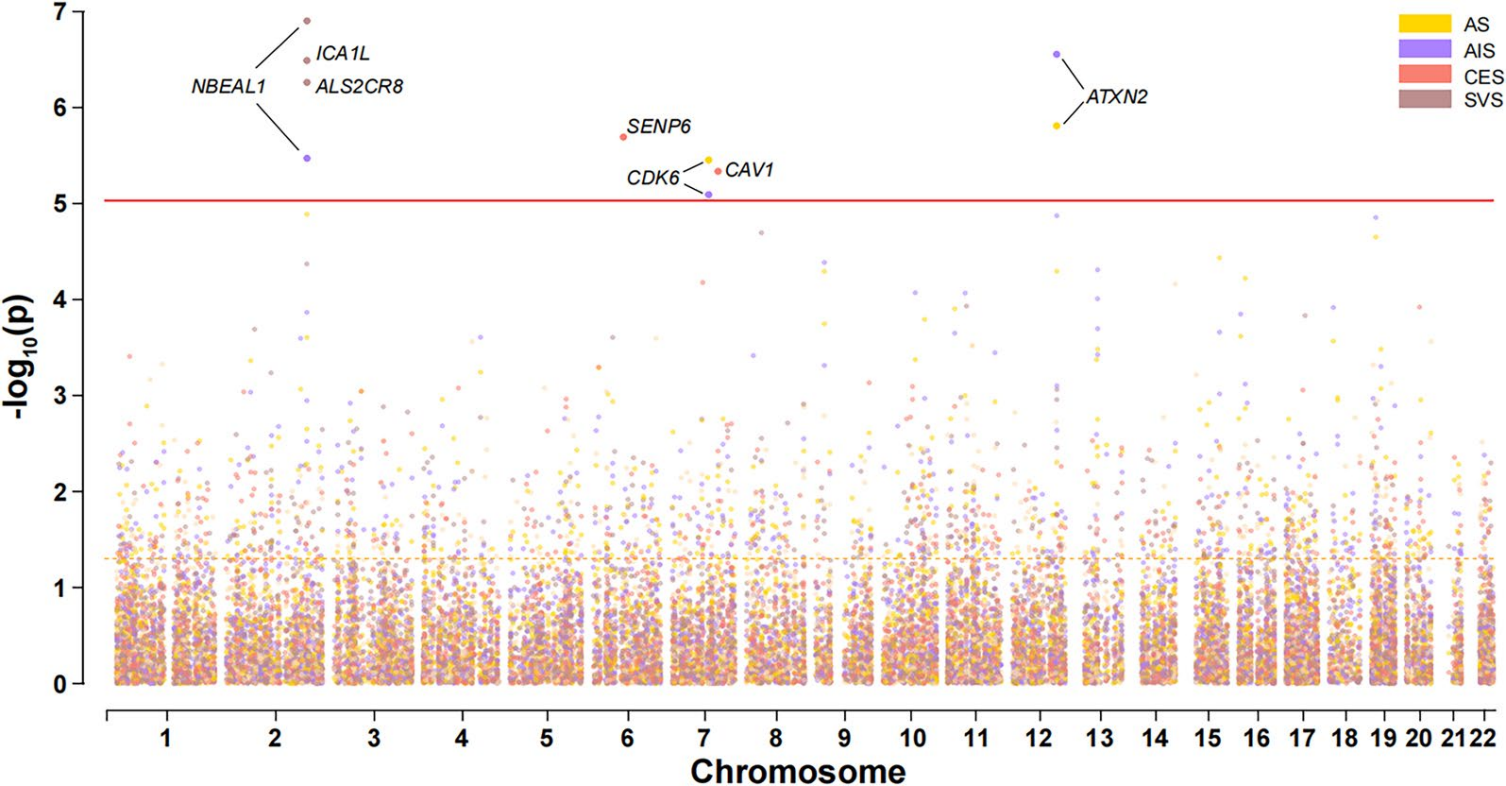
The Manhattan plot for the TWAS of stroke and stroke subtypes. The Manhattan plot shows the genes identified using TWAS for AS, AIS, LAS, CES, and SVS, respectively. The red line indicates the significant threshold for Bonferroni correction (*P* < 0.05/5371). The orange dash line indicates the nominal significance level (*P* < 0.05)

We then performed TWAS of stroke to provide insight into transcription level using data generated from dPFC (CommonMind Consortium; n = 452) [20]. There were 7 genes whose expression in brain were associated with stroke in TWAS (*P* < 0.05/number of genes analyzed) (Fig. 3 and Table 2). TWAS identified *ATXN2* (AS: Z-score: 4.805, *P* = 1.55×10^−6^; AIS: Z-score: 5.137, *P* = 2.79×10^−7^) and *CDK6* (AS: Z-score: − 4.638, *P* = 3.52 × 10^−6^; AIS: Z-score: − 4.463, *P* = 8.08 × 10^−6^) whose *cis*-regulated brain mRNA expression was both associated with AS and AIS, and the expression of *NBEAL1* (Z-score: 4.646, *P* = 3.38 × 10^−6^) was also associated with AIS (Additional file 1: Table S6 and S7). No significant association was found for *LAS* (Additional file 1: Table S8). As for CES, only *SENP6* (Z-score: − 4.751, *P* = 2.03 × 10^-6^) and *CAV1* (Z-score: − 4.582, *P* = 4.62 × 10^−6^) passed the Bonferroni correction (Additional file 1: Table S9). In the analysis for SVS, *NBEAL1* (Z-score: 5.286, *P* = 1.25 × 10^−7^), *ICA1L* (Z-score: 5.109, *P* = 3.24 × 10^−7^) and *ALS2CR8* (Z-score: 5.009, *P* = 5.47 × 10^−7^) showed evidence for association at the mRNA level (Additional file 1: Table S10). Interestingly, *ICA1L* was also significant in the SVS PWAS, suggesting that *ICA1L* regulates brain protein abundance via the regulation of brain mRNA expression.

**Table 2:** Candidate genes in brain associated with stroke and stroke subtypes using TWAS

Stroke subtype	Gene	CHR	Z-score	P-value
AS	*ATXN2*	12	4.805	1.55 × 10^–6^
	*CDK6*	7	− 4.638	3.52 × 10^–6^
AIS	*ATXN2*	12	5.137	2.79 × 10^–7^
	*NBEAL1*	2	4.646	3.38 × 10^–6^
	*CDK6*	7	− 4.463	8.08 × 10^–6^
LAS	–			
CES	*SENP6*	6	− 4.751	2.03 × 10^–6^
	*CAV1*	7	− 4.582	4.62 × 10^–6^
SVS	*NBEAL1*	2	5.286	1.25 × 10^–7^
	*ICA1L*	2	5.109	3.24 × 10^–7^
	*ALS2CR8*	2	5.009	5.47 × 10^–7^

### *ICA1L* was causally associated with SVS using MR in brain

MR analysis of brain pQTL and stroke GWAS identified 2 protein biomarkers that provided strong evidence of associations [*P* < 8.224 × 10^–5^ (0.05/608)] (Table 3). Genetically predicted higher ALDH2 was associated with higher AS and AIS risk (AS: OR [95% CI] 1.89 [1.41, 2.54]; AIS: OR [95% CI] 2.11 [1.53, 2.91]) and both of them were replicated in PWAS (Additional file 1: Table S11 and 12). However, no evidence was found between protein biomarkers and LAS or CES (Additional file 1: Table S13 and S14). As for SVS, the association between lower ICA1L and higher SVS risk was identified (OR [95% CI] 0.08 [0.03, 0.25]), which was also replicated in PWAS (Additional file 1: Table S15). Steiger filtering showed direct causal associations from changes of ALDH2 to development of AS and AIS (AS: Steiger P Value: 8.78 × 10^–14^; AIS: Steiger P Value: 9.43 × 10^–14^) and ICA1L to SVS (Steiger P Value: 1.22 × 10–^13^) (Table 3).

**Table 3:** Candidate genes identified by Mendelian randomization, Bayesian colocalization and Steiger filtering analysis in brain

Data source	Stroke subytpe	Gene	Beta	SE	P-value	PPH4	Correct Direction	Steiger_P Value	If replicated in PWAS/TWAS
pQTL	AS	*ALDH2*	0.639	0.150	2.06E−05	60.20%	TRUE	8.78E−14	Yes
	AIS	*ALDH2*	0.748	0.164	5.00E−06	17.30%	TRUE	9.43E−14	Yes
	LAS	–							
	CES	–							
	SVS	ICA1L*	− 2.521	0.569	9.49E−06	99.20%	TRUE	1.22E−13	Yes
eQTL	AS	*HDAC9*	0.253	0.041	5.19E−10	100.00%	TRUE	1.21E−09	No
	AIS	*HDAC9*	0.276	0.044	3.38E−10	100.00%	TRUE	1.24E−09	No
		*HECTD4*	0.242	0.050	1.44E−06	95.10%	TRUE	4.03E−09	No
	LAS	*HDAC9*	0.757	0.106	1.06E−12	100.00%	TRUE	1.59E−09	No
	CES	–							
	SVS	ICA1L*	0.229	0.046	5.73E−07	87.20%	TRUE	3.89E−36	Yes
		*CARF*	0.280	0.057	7.14E−07	81.60%	TRUE	3.79E−23	No
		*ADRB1*	− 0.359	0.075	1.47E−06	91.10%	TRUE	1.52E−33	No
		*NBEAL1*	0.317	0.068	2.64E-06	79.40%	TRUE	7.87E−17	Yes

Table shows the beta, SE and P values for the MR analysis of brain pQTL (above) and eQTL (down). PPH4 denotes the posterior probability that two traits share a causal genetic variant using Bayesian colocalization analysis. Correct Direction and P value are given for Steiger filtering analysis, which shows the correct direction for the effect between exposure and stroke risk in this table. * indicated the gene which was identified in the MR analysis using both pQTL and eQTL data

To figure out whether genes with evidence for being causal in stroke at the protein level were also relevant to stroke at the transcriptional level, we conducted MR analysis using brain eQTL data (Table 3). *HDAC9* gene displayed robust causal evidence with AS, AIS, and LAS in the MR (AS: OR [95% CI] 1.29 [1.19, 1.39]; AIS: OR [95% CI] 1.32 [1.21, 1.44]; LAS: OR [95% CI] 2.13 [1.73, 2.63]) (Additional file 1: Table S16, 17 and S18). Nevertheless, no association was found between the expression level and CES (Additional file 1: Table S19). Notably, 4 genes were associated with SVS, of which *ICA1L* and *NBEAL1* were also replicated in TWAS (Additional file 1: Table S20). All genes identified in the MR analysis passed Steiger filtering analysis (Table 3).

### Colocalization between stroke risk genes and p/eQTL in brain

We examined the posterior probability for a shared causal variant between a pQTL and stroke for the genes which met the Bonferroni-corrected *P* value threshold in previous MR analysis. However, only *ICA1L* met the criterion (PPH4 > 75%) in the analysis of SVS, indicating a shared single variant with SVS (Table 3). At transcriptional level, the colocalization analysis identified all the 6 genes which provided evidence of colocalization (PPH4 > 75%) (Table 3).

### Five genes were causally associated with stroke in blood

We investigated whether the genes associated with stroke in brain could be expressed through blood data. Applying MR to serum proteomic data, 4 genes with 8 significant causal associations with stroke survived from corrections for multiple testing methods and passed the Steiger filtering analysis (Additional file 1: Table S21). Of these, the concentration of MMP12 was inversely associated with AS, AIS, and LAS risk (AS: OR [95% CI]: 0.90 [0.87, 0.94]; AIS: OR [95% CI]: 0.89 [0.85, 0.93]; LAS: OR [95% CI]: 0.78 [0.70, 0.86]), while ABO was positively associated with AIS, LAS, and CES risk (AIS: OR [95% CI]: 1.03 [1.02, 1.05]; LAS: OR [95% CI] 1.09 [1.05, 1.13]; CES: OR [95% CI] 1.07 [1.04, 1.10]) (Additional file 1: Table S22–S25). As for SVS, results showed no gene survived corrections for multiple testing (Additional file 1: Table S26). We next repeated MR analysis using the whole-blood eQTL data. However, only CKAP2 displayed robust evidence with AIS in MR (Additional file 1: Table S27–3S1). It should be noted that those genes identified in blood were different from the genes identified in brains, indicating a distinct pathogenic mechanism in blood.

### Summary findings

**Fig. 4:**
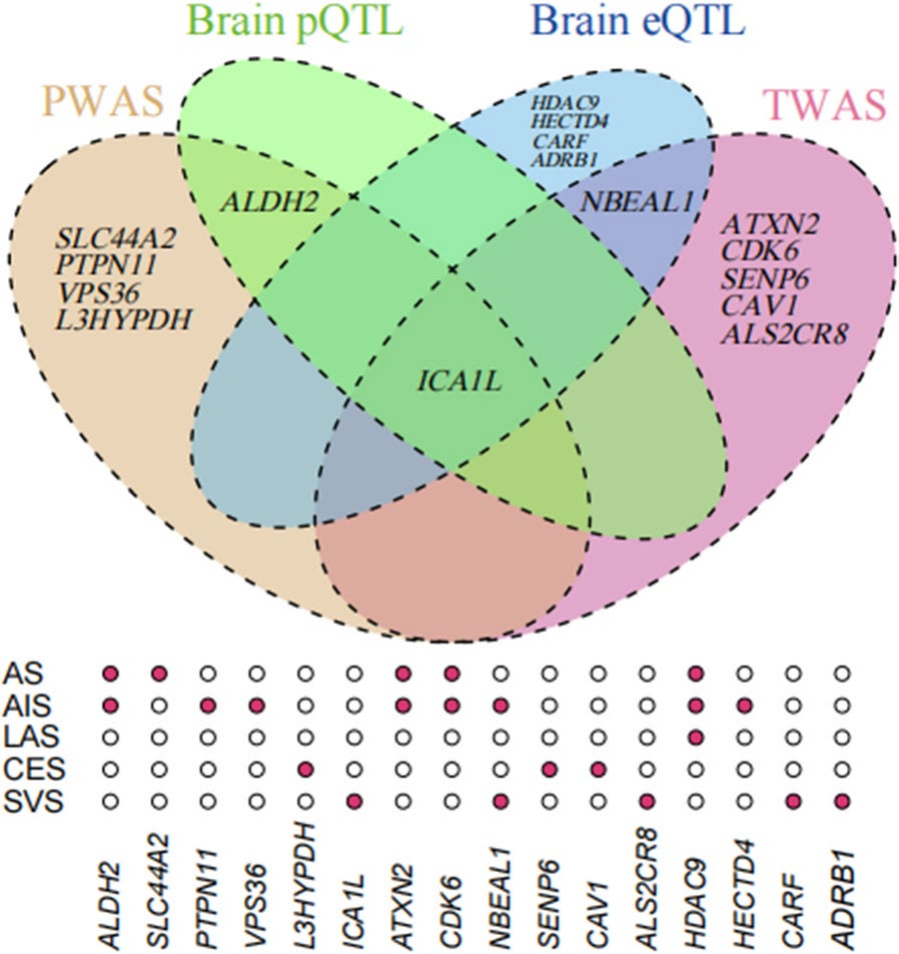
The Venn plots of the significant genes among four approaches for stroke and stroke subtypes. Brain pQTL and brain eQTL indicate the MR analysis using brain pQTL and brain eQTL data respectively. The red dots implicate where the significant associations are found for each gene among AS, AIS, LAS, CES, and SVS

Using PWAS, TWAS, MR and Bayesian colocalization analyses, *ICA1L* and *NBEAL1* were proved to be causal for stroke in brains (Fig. 4). Although there was evidence that *ALDH2* was associated with stroke in PWAS and MR, they did not reach the Bayesian colocalization’s criterion. Comparative analyses illustrated that there was no overlap between genes identified in brains and those in blood.

## Discussion

We conducted an integrative analysis of PWAS, TWAS, MR and Bayesian colocalization to identify causal genes for stroke and stroke subtypes using brain- and blood-derived data. Collectively, 16 genes were identified in brains, of which *ICA1L* showed causal for SVS at both protein and transcriptional level, while *NBEAL1* showed causal for SVS at transcriptional level only. Furthermore, 5 different genes (*MMP12*, *ABO*, *SCARF1*, *F11*, and *CKAP2*) were discovered in blood, which indicated two distinct pathogenesis for stroke in brain and blood.

Of the identified genes, *ICA1L* was previously discovered in a GWAS of cerebral small vessel disease [28] and its genetically elevated expression was associated with lacunar stroke in TWAS [29]. Employing a larger population of stroke in our pipeline, the results further verified the important role of *ICA1L* in SVS. ICA1L and its major binding partner PICK1 involved in cellular functions required dynamic remodeling of the actin cytoskeleton [30, 31], suggesting a potential role of actin cytoskeleton in the pathogenesis of SVS. However, it is worth noting that there was a diverse direction for *ICA1L* at protein and transcriptional level, and the expression in specific tissue may partly explain it. *ICA1L* mainly expresses in brain and testis tissues and its active participation in spermiogenesis has been identified [30]. Since stroke shows a marked sex bias with men having greater incidence of stroke [32], potential differential expression of *ICA1L* between male and female may account for this divarication.

By integrative analysis of TWAS, MR and colocalization analysis using brain-derived data, we prioritized another gene (*NBEAL1*)*,* which showed evidence of being associated with SVS. Neurobeachin-like 1 protein, encoded by *NBEAL1,* is one of the nine proteins that share a highly conserved domain known as the BEACH (Beige and Chediak-Higashi) domain [33]. *NBEAL1* have been detected previously in a GWAS of cerebral small vessel disease [28] and was found to affect cellular cholesterol metabolism and LDL uptake and was associated with coronary artery diseases [33], indicating that *NBEAL1* may influence the risk of stroke by LDL. Furthermore, a recent study also connected *NBEAL1* with white matter hyperintensity volumes [34]. And the highest lesion burden was found in patients with small artery occlusion [35], which pointed out the close relationship between *NBEAL1* and SVS. However, the biological functions of NBEAL1 in the pathogenesis of SVS is still elusive and need further investigations. Recalling the genes identified in each method, only a small part of genes was replicated in other approaches and this might be due to several reasons. Apart from the differences in statistical processing and measurement errors, the relationship between mRNAs and proteins could also be affected by difference in translation efficiency, protein degeneration, contextual confounds and protein-level buffering [36]. Furthermore, the sample sizes and number of genes analyzed in the transcriptome and proteome data varied considerably. Although the rest of 16 causal genes identified by the integrative analysis were only replicated in one or two analyses, some of them (e.g. *ALDH2*, *CDK6*, *HDAC9*, and *SLC44A2*) were previously reported linked to stroke, which also demonstrate the reliability of our integrative analysis in a way. *ALDH2,* which is associated with a poorer functional outcome of ischemic stroke [37] and increases the risk and susceptibility to hypertension or diabetes [38], has been found to protect against stroke by clearing the toxic aldehydes, for example, 4-hydroxy-2-nonenal (4-HNE) [39]. However, in the Bayesian colocalization analysis, there was insufficient evidence to show the abundance of ALDH2 and stroke shared common causal variants (PPH4 < 75%). The causal relationship between *ALDH2* and stroke still need further study. *CDK6* has been identified as a key regulator of atherosclerosis for *CDK6* knockdown can suppress proliferation of HASMC and HUASMC [40]. A previous study has also shown the down-regulation of *CDK6* in the penumbra surrounding the infarction region comparing with control [41], which supports the inverse association between expression of *CDK6* and the risk of AS and AIS in our TWAS analysis. In MR analysis, we found a causal relationship between an increased expression of *HDAC9* and the higher risk of AS, AIS, and LAS. *HDAC9*, whose enhanced expression is associated with increasing calcification and decreasing contractility in human aortic vascular smooth muscle cells [42], has also been identified in a GWAS for large vessel stroke [43]. Likewise, the *SLC44A2* rs2288904-A polymorphism showed protective effect in venous thrombosis [44], implicating its potential role in stroke by modulating thrombosis. Collectively, although there is relatively insufficient evidence for the associations of these genes with stroke in our integrative analysis, some of them have been reported to play an important role in the pathogenesis of stroke, which deserves further replication in population with larger sample size.

Using blood as a surrogate has been widely found to establish associations with brain-related traits. And a previous study has found that there were strong correlations between brain and blood (r_b_ ≥ 0.7) from cis-eQTL or mQTL data [45]. In our MR analysis between levels of blood proteomes and stroke risk, we found 4 genes (*MMP12*, *SCARF1*, *ABO*, and *F11*), of which *MMP12*, *ABO*, and *F11* were replicated, compared with a previous study that analyzed the association of circulating biomarker levels with stroke and stroke subtypes using a different study sample [46]. These candidate genes identified in blood are different from those discovered in brains. Similarly, in a recently published research, MR was used to analyzed the causal effect of pQTL data derived from CSF, plasma, and brain with seven neurological traits [19]. However, there was no overlap between results in plasma and brain for stroke after multiple testing correction [19]. The biological mechanisms of these genes with stroke pathogenesis reported already can partly explain this difference. ApoE/MMP-12 double knockouts mice showed reduced and more stable plaques in the brachiocephalic artery [47], indicating that MMP12 might participate in the stability of plaques [48]. A previous study reported an increased risk of thrombosis with the non-O blood groups (A, B or AB) [49], and this effect was hypothesized to affect von Willebrand Factor (vWF) clearance [50]. Similarly, genetic variation in *F11* was also associated with both deep vein thrombosis and the level of coagulation factor XI [51]. Taken together, these genes identified in MR from blood proteomes mainly focus on coagulation and atherosclerosis, which finally cause stroke. Besides, given that different tissues have different expression profiles, the differences among methods of tissue collection, extraction and analysis further lead to only a small overlap of genes derived from brains and blood.

In general, our study has a key advantage-we integrated multidimensional QTL data to provide comprehensive insights into complex biological systems of stroke from both brains and blood. Comparatively, using single method or single dimensional data (e.g. TWAS and MR) to identify stroke candidate genes has some limitations. First, methodologically, the accuracy of TWAS relied on training cohort size and the quality of the training data [8]. As for MR, the power of MR depended on the proportion of total variance of the exposure explained by the genetic variants and the strength of the causal association between exposure and outcome [52]. Second, dimensionally, only using single dimensional data (protein or transcriptional level) to identify the underlying genes for stroke pathogenesis is insufficient, leading to false positive results. Therefore, our integrative analyses bring data from genome, transcriptome, and proteome together through multi approaches, and contribute to identifying the key causal genes in the pathogenesis of stroke. Besides, two genes (*SLC25A44* and *LRCH1*), whose expression were significantly associated with stroke after Bonferroni correction, were identified as candidate genes in a recent TWAS in adipose [53]. These genes were not discovered in our integrative analysis, indicating a tissue-specific expressional pattern. Using the directly related brain tissue, our analysis could prioritize candidate genes more relevant with stroke.

There are also some limitations in our study. First, the genes tested in PWAS were relatively smaller than those tested in TWAS, resulting in fewer genes identified in the two analyses. And this can be addressed by using a larger brain sample dataset. Second, gene expression is a highly complicated process, varying in time and space. Our study only investigated the candidate genes in brains and blood, future work may focus on other tissues. Third, we only performed the analysis in one dataset for each stroke subtype, which needs further validation using larger stroke GWAS datasets. Nevertheless, it is worth noting that some genes were replicated in analyses for several stroke subtypes, which validated their potential role to some degree. Fourth, it is insufficient to elucidate the numerous stroke GWAS-identified loci from protein and transcriptional level. Methylation data can be integrated into the analysis to reach a more comprehensive understanding of disease progression. Fifth, our study mainly focused on European subjects, and it should be careful to extend our results to other ethnicities. In addition, functional genomic approaches and biological experiments are necessary to understand the complex biology of stroke and illustrate the molecular mechanisms behind.

In conclusion, this integrative analysis identified *ICA1L* and *NBEAL1*, whose expression and protein abundances are associated with the risk of small-vessel stroke. Our study offered hints for future biological and therapeutic studies to identify their potential roles in stroke pathogenesis.

## Supplementary Information

**Additional file 1:** Supplementary Tables 1–32.

## Data Availability

The data of brain pQTL from the Religious Orders Study/Memory and Aging Project (ROS/MAP) study is available through https://doi.org/10.7303/syn23627957. Above data from ROS/MAP is available for general research use according to the following requirements for data access and data attribution (https://adknowledgeportal.org/DataAccess/Instructions). The data of brain eQTL from the PsychENCODE Consortium are accessible in BESD format through https://cnsgenomics.com/software/smr/#eQTLsummarydata. The data from AGES Reykjavik study is available from www.sciencemag.org/cgi/content/full/science.aaq1327/DC1. The data from the AGES Reykjavik study was acquired through collaboration (AGES_data_request@hjarta.is) under a data usage agreement with the IHA. GTEx can be accessed at https://gtexportal.org/home/datasets (GTEx Analysis V6). The GWAS datasets of the stroke analyzed during the current study are available from https://www.megastroke.org/.

## Abbreviations

4-HNE: 4-Hydroxy-2-nonenal
AIS: Any ischemic stroke
BH: Benjamini-Hochberg
CES: Cardioembolic stroke
dPFC: Dorsolateral prefrontal cortex
DALYs: Disability-adjusted life years
FUSION: Functional Summary-based Imputation
FDR: False discovery rate
GTEx: Genotype-Tissue Expression
GWASs: Genome-wide association studies
IVW: Inverse-variance weighted
LAS: Large artery stroke
LD: Linkage disequilibrium
MR: Mendelian randomization
MS: Mass spectrometry
PFC: Prefrontal cortex
PP: Posterior probability
PWASs: Proteome-wide association studies
QTL: Quantitative trait loci
ROS/MAP: Religious Orders Study/Memory and Aging Project
SNP: Single nucleotide polymorphisms
SOMAmer: Slow-Off rate Modified Aptamer
SVS: Small vessel stroke
TMT: Tandem mass tag
TWASs: Transcriptome-wide association studies
vWF: Von Willebrand Factor
